# Innate lymphoid cells and the skin

**DOI:** 10.1186/1471-5945-14-18

**Published:** 2014-11-26

**Authors:** Maryam Salimi, Graham Ogg

**Affiliations:** Department of Medicine, MRC Human Immunology Unit, NIHR Biomedical Research Centre, Radcliffe University of Oxford, Oxford, UK

**Keywords:** Innate lymphoid cells, Atopic dermatitis, Psoriasis, IL-33, IL-25, TSLP, PGD2, KLRG1, E-cadherin

## Abstract

Innate lymphoid cells are an emerging family of effector cells that contribute to lymphoid organogenesis, metabolism, tissue remodelling and protection against infections. They maintain homeostatic immunity at barrier surfaces such as lung, skin and gut (Nature 464:1367–1371, 2010, Nat Rev Immunol 13: 145–149, 2013). Several human and mouse studies suggest a role for innate lymphoid cells in inflammatory skin conditions including atopic eczema and psoriasis. Here we review the innate lymphoid cell family and discuss their function in the skin and during inflammation.

## Introduction

### Innate lymphoid cells

Recent advances in the field of immunology have identified a novel family of CD45 expressing haematopoietic effector cells. These cells have phenotypical features of lymphoid cells but lack rearranged antigen specific surface receptors of adaptive immune cells and are termed innate lymphoid cells (ILCs) [1]. ILCs are essential for lymphoid organogenesis, metabolism, tissue homeostasis and repair, protection against viral and helminth infections [2–4]. They reside in the blood, spleen, intestine, liver, fat associated lymphoid clusters (FALC), and mesenteric lymph nodes of humans and mice. Their development depends on the expression of the transcriptional repressor Id2 that regulates the activity of helix-loop-helix protein E47 and RORC. Cytokines that signal through the common γ chain of IL-2 receptor and Jak3 are essential for their maintenance.

ILCs are thought to be able to influence adaptive immune responses as they reside in the interface of T and B cell zones in the splenic follicles of mice and can express co-stimulatory molecules essential for T cell priming and survival, including CD40 ligand and CD30 ligand [5]. Each distinct functional subset produces cytokines that were previously thought to be specific to adaptive immune system lineages. Based on their cytokine profile and functional characteristics, they can be divided into three main groups [1], although recent studies on lineage relationships and common precursors of ILCs make this classification debatable [6].

## Review

### Group 1 ILCs

The most studied prototype of this family is group 1 ILCs including NK cells and group 1 innate lymphoid cells (ILC1) which were identified in 1975 and 2012, respectively [7–9]. They express transcription factors T-bet, Nfil3 (E4BP4) and Eomes [10], and IL-15 and IL-12 are required for their development and function. NK cell subsets include cytolytic effectors of the innate immune system and can produce IFN-γ, TNF-α, MIP1-α, MIP1-β and RANTES. They are believed to be responsible for defence against intracellular pathogens, tumours and viruses, but may contribute to aberrant inflammation in certain settings. Unlike NK cells, ILC1 lack granzyme B and perforin but express CD103 [11] and CXCR3 [9]. IL-7Rα^+^ IL-12Rβ2^+^ IL-1R^+^ group 1 ILCs produce IFN-γ in response to IL-12 and are enriched in inflamed mucosal tissue such as tonsils, intestine and in diseased tissue including the lamina propria of patients with Crohn’s disease [11] and inflamed lungs of patients with chronic obstructive pulmonary disease (COPD) [12]. Interestingly group 1 ILCs are absent from foetal gut and develop after colonisation of the intestine with commensal bacteria [9]. These findings indicate their potential role in protection against certain bacteria in homeostatic conditions and their involvement in the pathogenesis of inflammatory bowel disease and COPD. The origin of group 1 ILC is not clear; however several studies have showed that RORɣt– IFN-ɣ producing ILC1 can originate from NKp44^+^ group 3 ILC under the influence of IL-12 and IL-15 [9, 13].

### Group 2 ILCs

The production of key type 2 cytokines, IL-13, IL-4 and IL-5 in response to epithelial cytokines IL-25 and IL-33 in Rag^−/−^ mice led to the discovery of group 2 ILCs. In 2010, three separate groups reported lineage negative cells (CD3, CD4, CD8α, TCRαβ, TCRɣδ, CD5, CD19, B220, NK1.1, Ter119 (Ly76), Gr-1 (Ly6g), Mac-1 (Itgam), CD11c (Itgax) and FcϵRIα) that expressed c-Kit (CD117) and T1/ST2, CD90 (Thy-1), CD45 and IL-7Rα (CD127) in mice. They were designated nuocytes [2], innate helper type 2 (IHC) [14] and natural helper cells (NHC) [15] but demonstrated similar functional characteristics. Concurrently, Saenz described a similar population of multi-potent progenitor type 2 (MPP type2) cells but unlike other populations, MPP type 2 cells exhibited progenitor capacity and could differentiate to myeloid and lymphoid lineage descendants [16]. Therefore, it has been speculated that they might be precursors of group 2 ILCs [17]. Recently the term ILC2 was proposed to group type 2 cytokine producing ILC in to a single family [1]. Lack of RORγt expression and IFN-ɣ production differentiate this group of innate cells from LTi cells and ILC1, respectively. ILC2s were described in fat associated lymphoid clusters (FALC), mesenteric lymph nodes (mLN), intestine and gut associated lymphoid tissues (GALT), liver and spleen. Bearing IL-17RB (IL-17BR, IL-25R), ST-2 (IL-33R) and TSLP receptors ILC2 cells respond to epithelial cytokines including IL-25 (IL-17E), IL-33 and TSLP, by producing type 2 cytokines as IL-13, IL-4, IL-5, IL-9.

Human ILC2s were discovered in healthy human lung parenchyma and broncho-alveolar lavage (BAL) fluid of patients receiving a lung transplant as lineage negative cells (CD3, TCRαβ, CD11c, CD11b, CD56, CD19) that express IL-7Rα and ST2 subunit of the IL-33 receptor [18]. Spits *et al*. reported lineage negative (CD3, CD4, CD11c, CD14, CD19, CD34, CD123, TCRαβ, TCRɣδ, BDCA2, and FcϵRI), CD45^hi^, CD127^+^ and CD117^+^ cells in peripheral blood, foetal gut and inflamed nasal polyps of patients with rhinosinusitis. They also express prostaglandin D2 receptor (CRTH2), CD161 (KLRB1), CD7 and CD25. In response to epithelial cytokines and IL-2 the cells produce large amounts of IL-13 and IL-5 but not IL-17A or IL-22 [19, 20].

ILC2 represent a vital source of IL-13 for expulsion of the gut helminth, *Nippostrongylus brasiliensis* [2, 15, 16] by inducing goblet cell hyperplasia, eosinophilia and intestinal smooth muscle cell contraction. ILC2 also contribute to homeostasis and allergic responses in the airways. Using IL-4^+/eGFP^ IL-13^+/Tom^ dual reporter mice, it was shown that ILC2s were the major source of type 2 cytokines in Ovalbumin induced allergy and after intranasal administration of IL-25 and IL-33 [21]. Moreover, depletion of lung resident ILC2 in mice after infection with H1N1 influenza virus A resulted in impaired airway epithelial integrity and lung function, with exaggerated thermodysregulation and higher total protein concentration in the broncho-alveolar lavage (BAL) fluid. Such function is predominantly mediated by amphiregulin production by ILC2, a wound-healing modulator of the epidermal growth factor family [18]. Recent studies demonstrated mutual interaction between ILC2 and T cells [22, 23]. Activated T cells produce IL-2 that induces proliferation and cytokine production of ILC2 [23]. ILC2 in humans and mice express MHC-II and co-stimulatory molecules CD80 and CD86. Co-culture of ILC2s and T cells in the presence of antigen, induced TH2 differentiation and type 2 cytokine production in T cells [22, 23]. In addition, ILC2 also enhance differentiation of polyclonally activated naïve T cells to a TH2 phenotype in an MHC-independent contact-dependent manner [23]. Furthermore, *N. brasiliensis* infection of two mouse models deficient in ILC2 showed delayed worm expulsion and a dramatic decrease in IL-13 and IL-5 producing CD4^+^ T cells. Moreover, IL-2 released from T cells promotes ILC2 proliferation and cytokine production [22].

### Group 3 ILCs

Group 3 ILCs include lymphoid tissue inducers (LTi cells), NCR^+^ ILC3 (NK22, NCR22, ILC22) and NCR^−^ ILC3 (ILC17) [24]. Group 3 ILCs are important in inflammation, anti-microbial protection, mucosal immunity and homeostasis. LTi cells express c-Kit, IL-7Rα, IL-1R, IL-23R and lymphotoxin-β (LTβ), CCR6, and aryl hydrocarbon receptor (AHR) [25]. They initiate lymphoid structure formation and induce expression of VCAM-1 and ICAM-1 on mesenchymal cells through LTβR and TNFR signalling during embryogenesis and produce IL-17A [26]. After birth, LTi cells contribute to the formation of solitary lymphoid follicles and Peyer’s patches. After birth splenic LTi cells produce IL-22 and IL-17A [25] in response to yeast cell wall product zymosan which suggests that they contribute to host defence. Furthermore, they also support class switching to IgA and are thus important for adaptive immune responses [27].

Two phenotypically distinct RORɣt dependent subsets of ILC3 were characterised recently, and based on the expression of natural cytotoxicity receptors, NKp46 in mice and NKp44 in human, they were divided into NCR^+^ [24] and NCR^−^ ILC3 [1]. Postnatal NCR^+^ ILC3 derived from tonsils largely produce IL-22 and small amounts of IL-17. They are thymus independent and reside in the intestine, dermis, tonsils and mLNs. NKp44^+^ RORɣt^+^ IL-22^+^ are diminished in germ free mice which suggests that their maintenance and functional properties are largely dependent on commensal bacteria [28]. IL-22 produced by RORɣt^+^ ILCs is essential in protection against *Citrobacter rodentium* induced acute colitis in mice [28]. Recently it was shown that Notch-2-dependent CD103^+^ CD11b^+^ cDCs are a major source of IL-23 during early stages of infection with *Citrobacter rodentium*. The population expansion of these cells was mediated by LTβR signalling [29]. The expression of IL-22 in NCR^+^ ILC3 is negatively regulated by the epithelial cytokine IL-25 as intestinal inflammation and epithelial damage by administration of dextran sodium sulfate (DSS) that concomitantly increased IL-23 and reduced IL-25, induced population expansion and IL-22 production by RORɣt^+^ILCs [30]. The NCR^−^ ILC3 population in mice expresses SCA-1, IL-23 receptor, transcription factor RORɣt and high levels of Thy-1. The cells accumulate in the gut during *Helicobacter hepaticus* induced colitis. Stimulation of NCR^−^ ILC3s with IL-23 induces production of IL-17 and IFN-ɣ [3].Recent studies showed that group 3 innate lymphoid cells can regulate adaptive immune responses. NCR^−^ ILC3 express high levels of major histocompatibility complex class II (MHCII) whereas NKp46^+^ ILC3 express minimal levels of MHCII. Although ILC3 can process and present antigen, they cannot induce T cell proliferation of naïve T cells as they lack co-stimulatory molecules, CD80, CD86, and CD40. Instead they appear to reduce T cell responses to commensal flora. Lack of MHC II on RORɣt^+^ ILCs induces spontaneous mild colitis, splenomegaly, shortened intestine and crypt elongation [31].

### ILC2 origin and transcription factors

Group 2 innate lymphoid cells arise in bone marrow from common lymphoid progenitors (CLP) at the double-negative stage 1 (DN1) and stage 2 (DN2). Their presence in thymus deficient Foxn1^nu/nu^ (nude) mice confirms that they do not require the thymus for their development [20, 32]. Unlike most previous studies that categorized NK and LTi cells in group 1 and group 3 ILCs respectively, recent work by Constantinides *et al*. showed a common ILC progenitor (ILCP) in foetal liver and adult bone marrow that can differentiate into ILC1, ILC2 and ILC3 but not NK and LTi cells. ILCP were Lin^−^ IL-7Rα^+^ c-Kit^+^ α4β7^+^ and phenotypically similar to precursors of LTi cells. High levels of PLZF, a transcription factor associated with NKT cells, as well as high levels of Id2, GATA3 and TOX were found in ILCP. PLZF^+^ ILCP arise from an α4β7 IL-7Rα^+^ population from which NK and LTi progenitors are also known to develop. However, interestingly depletion of PLZF altered ILC development but did not affect NK and LTi cells, which shows that NK and LTi cells have distinct precursors [6].

ILC2s require IL-7, IL-33 and signalling through tyrosine kinase receptor Flt-3 [32]. Jak3^−/−^ mice are deficient of ILC2 which confirms their requirement for signalling through common γ chain of IL-2 [20]. T cell factor-1 (TCF-1) essential for normal T cell lineage specification and is also required for development of ILC2. In Tcf7^−/−^ mice the frequency of ILC2 is 5% of wild type mice, and the remaining ILC2 are functionally compromised. Transient Notch signalling is another vital requirement for ILC2 development that acts upstream of TCF-1 but does not require the HES-1 pathway [33]. Forced expression of TCF-1 can bypass Notch requirement by up-regulating GATA-3 expression. GATA-3 maintains the expression of IL-17RB, IL-2R, IL-1RL1 receptors and production of IL-13 and IL-5. Its effect is intrinsic and dose dependent [33, 34]. Upon activation with IL-33 or a combination of IL-2 and IL-25, it binds to the IL-5 and IL-13 promoter in a p38 dependent manner [35]. GATA-3 induces the expression STAT-5 and increases responsiveness to IL-33 and TSLP [36]. Unlike TH2 cells, the expression of GATA3 and production of type 2 cytokines in the lung resident ILC2 in mice are STAT-6 and STAT-3 independent [37]. STAT-6 regulates the proliferation of these cells and induction of eosinophilia in response to Alternaria challenge.

Consistent with the lymphoid origin of ILC2, the transcription factor Ikaros is necessary for their development [20]. Although in one report a trace of RAG-1 expression was detected in these cells, mature ILC2s do not express any rearranged antigen receptors [38] and unlike other members of ILC family, their development is largely dependent on RORA but not RORC expression [32]. The precise interaction between GATA-3 and RORA is not clear; Mjösberg *et al*. showed that RORA is not regulated by GATA-3 and these two transcription factors possibly work in parallel during development of ILC2, whereas Wolterink *et al*. observed the lack of RORα expression in the absence of GATA-3 [34, 36]. Although RORA and GATA-3 have a pivotal role in development of ILC2, RORA is not essential for cytokine production and maintenance of mature ILC2 [35]. Lung ILC2 were observed in germ free mice and therefore thought to be independent of commensal bacteria for their development [18]. Although a degree of plasticity has been shown in ILC3 and ILC1 populations as a proportion of RORɣt^+^ NKR^+^ LTi cells can down regulate RORɣt and produce IFN-ɣ [13], it has yet to be determined whether ILC2 have any plastic characteristics.

### ILC2 and skin

Kim *et al*. provided the first evidence on the presence of an ILC2 population in mouse and human skin. In mice, they reported a population of Lin^−^ CD25^+^ ST2^+^ c-Kit^+^ CD127^+^ ICOS^+^ that did not express ILC3 associated markers CD4, NKp46 and RORɣt. Although they found a similar population in healthy human skin (Lin^−^ CD25^+^ IL-33^+^) these cells were negative for CRTH2 and CD161, previously described markers of ILC2 in humans [19], and they only acquired the expression of these markers in atopic dermatitis (AD) lesions. Therefore it raised the possibility that this population is either a distinct population of ILCs in human skin or is in a different stage of activation.

**Figure 1:**
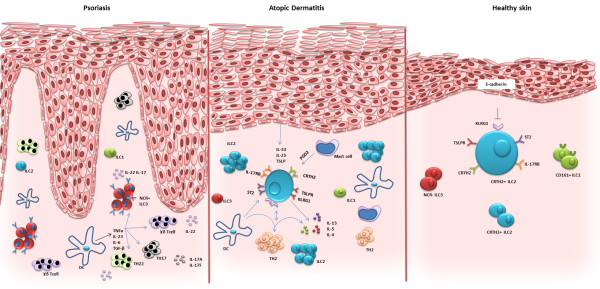
**ILC1, ILC2 and ILC3 interactions in human skin.** ILC1s express CD161. ILC2s express IL-17RB, ST2, CRTH2, TSLPR and an inhibitory receptor KLRG1. In homeostatic conditions, the expression of adhesion molecule E-cadherin on normal human keratinocytes inhibits the activation of ILC2s. NKp44^−^ ILC3s are the main subset of ILC3 in healthy skin. ILC2s are enriched in atopic dermatitis lesions and show higher expression of ST2, IL-17RB and TSLP-R, probably an activated phenotype. They express IL-13, IL-5, and IL-4 in response to IL-33, IL-25 and TSLP produced by keratinocytes and PGD2 released by mast cells and other cells. Concurrently, the diminished expression of E-cadherin on keratinocytes is a novel mechanism of sensing a dysfunctional barrier. The frequency of ILC1 and ILC3 in AD lesions are similar to healthy skin. The frequency of NKp44^+^ ILC3s is increased in psoriatic skin lesions. They produce IL-22 when stimulated with IL-23.

Later, the existence of ILC2 in human skin with the same morphology as described in other organs was confirmed [39–41]. Skin resident ILC2 did not express common lineage markers (CD3, CD8, CD14, CD19, CD56, CD11c, CD11b, FcεRI, TCR-αβ, TCR-ɣδ and CD123) but were positive for CD45, IL-7Rα, CRTH2, CD161, c-Kit, CD25 and ICOS [39, 40]. They expressed transcription factors RORα and GATA-3 whereas no RORɣt was detected [39]. ILC2 isolated from peripheral blood express skin homing markers cutaneous lymphocyte antigen (CLA), CCR10 [41] and the levels were further up regulated in the skin resident ILC2 [39] (16.7% to 37% and 0.9% to 84%, respectively) which suggests that similar to other organs, bone marrow derived ILC2 circulate in the blood and can migrate to the skin. ILC2 effector functions are at least partly mediated through cytokine receptors IL25R (IL-17RB), IL-33R (ST2) and TSLP-R [39–42] (Figure 1). In an activated state, these receptors are further up-regulated. IL-33 alone or in combination with IL-25 and TSLP could increase IL-13 and IL-5 production in human skin resident ILC2 [39]. In another report the combination of IL-2 and TSLP induced production of type 2 cytokines and addition of IL-25 to this cytokine mixture further enhanced IL-13 production, indicative of a synergistic effect. This synergy was not observed with IL-33 [41]. Activation of skin resident ILC2 promotes amphiregulin production, which is a ligand for epithelial growth factor receptor (EGFR) and regulates proliferation, apoptosis and migration of epithelial cells, and therefore contributes to wound healing and tissue repair [39].

Roediger *et al*. demonstrated potential immunosurveillance activity of ILC2 in mouse skin. They reported a unique and abundant population of CD45^+^ CD11b^−^ CD90^hi^ CD3^−^ CD2^−^ c-Kit^−^ IL-17RB^+^ ILC2s in the dermis of naïve mice and called them ‘dermal ILC2’ (dILC2). Dermal ILC2 expressed integrin αEβ7 (CD103) and comprised 5-10% of CD45^+^ cells. Using 4C13R dual reporter mice in which single alleles of IL-13 and IL-4 were substituted with dsRed and AmCyan respectively, they showed that homeostasis of the skin in the steady state was mainly controlled by CD3^−^ NK1.1^−^ dermal ILC2 rather than TH2 cells as they were the main producers of IL-13. Although the dermal ILC2 population was unable to produce IL-4 under homeostatic conditions, the cells acquired this capacity upon stimulation with TSLP. Furthermore, intravital multiphoton microscopy showed that CXCR6^+^ ILC2 are mainly aggregated in the close vicinity of blood vessels. They constantly patrol the skin local microenvironment with rapid migration (5 μm/min) but with intermittent long interactions with dermal mast cells that lasted 20 to 30 minutes. Interestingly mast cells were not essential for development and maintenance of dermal ILC2 as mast cell deficient mice (B6-Kit^W−sh/W−sh^ and WBB6F1-Kit^W/W−v^) had intact population of dermal ILC2, but in the presence of mast cells, dermal ILC2 were able to regulate them through IL-13.

Anti-CD90.2 antibody is routinely used to deplete ILC2 in mice. This method has been successfully used in the skin by Salimi *et al*. [39] and Kim *et al*. [42] although Roediger *et al*. [20] could not deplete CD103^+^ dILC2 from the skin using this method and only the ILC2 population in the spleen was depleted, which suggests that CD103^+^ dILC2 population might represent a distinct sub-population of ILC2 in the skin. Therefore instead of using a depletion strategy to study ILC2 function in the skin, they activated dILC2 *in vivo* using complexes of IL-2 and JES6-1. Consistent with their immunomodulatory function, dILC2 underwent proliferation and produced large amounts of IL-13 and IL-5. IL-13 suppressed the IgE dependent release of inflammatory cytokines by mast cells in a dose dependent manner [20].

The interaction between ILC2 and mast cells is important in regulating allergic type responses. Human ILC2 can respond to prostaglandin D2 (PGD2), a major metabolite produced by mast cells and other cells. This interaction is mediated through CRTH2 [40, 43]. Activation of human ILC2 by PGD2 increased expression of type 2 cytokines as well as IL-3, IL-8, IL-9, IL-21, GM-CSF and CSF-1. Interestingly PGD2 can induce production of IL-4 in human ILC2 which was not observed following stimulation with IL-25 and IL-33 [39–41]. PGD2 stimulation can augment expression of IL-33 receptor (ST2) and the IL-17A subunit of IL-25 receptor [40] and enhance ILC2 responses to IL-33 and IL-25 [43]. Consistent with their quick effector function, both PGD2 and IL-33 induced rapid migration of ILC2. IgE activated mast cell supernatant which contained endogenously synthesized PGD2, mediated the same CRTH2-dependent effector functions such as cell migration and cytokine production in human ILC2 as exogenous PGD2.

### Innate lymphoid cells, atopic dermatitis and psoriatic skin inflammation

Atopic dermatitis is a chronic, relapsing inflammatory skin condition characterized by hypersensitivity reactions to common environmental allergens. Given increased levels of type 2 cytokines, IL-13, IL-4 and IL-5, in acute atopic eczema lesions and enhanced production of epithelial cytokines IL-33, IL-25 and TSLP, it is plausible that group 2 innate lymphoid cells contribute to the pathogenesis of atopic dermatitis. Indeed, ILC2 were found in higher proportions in the lesions of patients with atopic dermatitis while the frequency of the cells was similar in the blood of both healthy and atopic individuals [39, 42]. ILC2 in atopic lesions showed an activated phenotype with higher expression of cytokine receptors, IL-17B (IL-25R), ST2 (IL-33R) and TSLP receptor. Increased mRNA levels of IL-17RB, ST2, TSLPR, CRTH2, RORA and AREG were detected in lesional skin biopsies of AD patients [39]. Interestingly sampling the skin with or without intra epidermal delivery of house dust mite (HDM) using a suction blister technique in humans, showed that ILC2 infiltrate in to the skin in response to allergen challenge in allergic individuals. A similar effect was seen after subcutaneous administration of HDM extract in mice.

A possible mechanism of skin barrier sensing was described for activated ILC2 in atopic dermatitis lesions. ILC2 express the KLRG1 receptor that upon interaction with its ligand, E-cadherin, conveys inhibitory signals. KLRG1 is up-regulated in response to ILC2 stimulating cytokines. KLRG1 ligation to E-cadherin in a plate-bound assay reduced production of type 2 cytokines, IL-13 and IL-5, and diminished amphiregulin expression and decreased GATA-3 expression. It is speculated that in healthy tissues expressing E-cadherin, ligation to KLRG1 regulates ILC2 responses. There is known to be a significant down-regulation of E-cadherin on keratinocytes in AD lesions which may reduce inhibitory signals to ILC2 and lead to production of type 2 cytokines (Figure 1). The type 2 cytokines may contribute to cutaneous inflammation by down-regulating filaggrin and anti-microbial peptides [44].

Group 2 innate lymphoid cells contribute to inflammation in mouse models of AD-like inflammation. hK14mIL33tg mice in which IL-33 is expressed under the keratin 14 promoter developed spontaneous atopic dermatitis-like inflammation of the skin at 6–8 weeks of age in specific pathogen-free (SPF) conditions. Similar to AD lesions, the pattern of IL-33 expression in this model was confined to the epidermal cell nuclei. Dermatitis lesions in hK14mIL33tg mice were associated with a substantial increase in the concentration of IL-13, IL-5, RANTES/CCL5 and Eotoxin 1/CCL11, whereas the levels of TSLP, IFN-γ and TNF-α were not altered. Increased degranulating IgE^+^ c-Kit^+^ mast cells as well as a significant infiltration of eosinophils were observed in the lesions and blood of transgenic mice (7.4 and 4.5 fold higher expression compared to wild type mice respectively). Concurrently skin lesions and regional lymph nodes were enriched for Lin^−^ ST2^+^ Sca-1^+^ ILC2 producing IL-5 and IL-13 [45].

Interestingly treatment of Rag1^−/−^ mice with IL-2 and anti-IL-2 complex (JES6-1) for 2–3 weeks induced spontaneous skin inflammation around ears, eyes, mouth and tail. Extensive accumulation of neutrophils, eosinophils and increased degranulation of mast cells were observed in these lesions which were caused in part by dILC2. dILC2 showed an activated phenotype with higher expression of CD25, ICOS, CD69, ST2 and enhanced production of IL-13 and IL-5 [20].

Topical application of a form of vitamin D3, calcipotriol (MC903), induces ear thickening, xerosis and histopathological changes comparable to AD lesions and is recognized as an experimental murine model of atopic dermatitis. Coincident with ear thickening, increased infiltration of ILC2 was observed in the ear pinna and draining lymph nodes of treated mice [39, 42] which was independent of adaptive immunity as Rag1^−/−^ mice still developed AD-like inflammation. Treatment of Rorc^−/−^ mice with calcipotriol induced the same level of inflammation which ruled out the involvement of RORɣt dependent ILC3s [42]. Depleting ILC2 using anti-CD90.2 [39, 42] or anti-CD25 antibodies [42] in Rag1^−/−^ mice significantly ameliorated inflammation and histopathological changes observed in this model. To eliminate the possibility of involvement of other cell types that potentially express CD90.2 antigen, Salimi *et al*. used RORα^−/−^ bone marrow chimera mice that had significantly lower numbers of ILC2s. Absence of ILC2s strongly correlated with reduced ear swelling in this model [39].

The hierarchal significance of ILC2 inducing epithelial cytokines IL-25, IL33 and TSLP was established using IL-17RB^−/−^ IL-1RL1^−/−^ and TSLPR^−/−^ respectively backcrossed to BALB/c and C57BL/6 strain backgrounds. In cytokine receptor deficient mice generated on a BALB/c strain background, the greatest protection against calcipotriol induced inflammation was observed in mice lacking IL-25 signalling pathway followed by IL-1RL1 deficient mice. The reduction in ear swelling correlated with a decreased frequency of ILC2s in ear pinna and draining lymph nodes. TSLPR deficient BALB/c mice showed a modest reduction in ILC2 numbers and ear inflammation. Surprisingly, the C57BL/6 strain background showed greater dependency on TSLP signalling pathway with a lower but significant role for IL-25 and IL-33 signalling pathways in inducing AD-like inflammation compared to the BALB/c background [39].

It is noteworthy to mention that as well as ILC2s, populations of NCR^−^ ILC3 (26.5 ± 8.6%) and CD161^+^ ILC1 (24.8 ± 11.0% of CD45^+^ Lin^−^ CD127^+^ ILC) were observed in normal human skin [41] (Figure 1). There is a noticeable population of NKp44^+^ ILC3s in cultured dermal explants, although rare in freshly isolated cells. NCR^+^ ILC3s differentiated from NCR^−^ ILC3 upon culture with IL-23, IL-1β and express IL-22.

Blood and lesional skin biopsies of patients with psoriatic skin inflammation showed enrichment of NCR^+^ ILC3 although similar frequencies of CD161^+^ ILC1 and CRTH2^+^ ILC2 were observed [41, 46, 47] (Figure 1). These cells were an innate source of IL-22. One report found that the frequency of NKp44^+^ ILC3 correlated with disease severity using PASI score [41] whereas another report could not show a similar finding [46]. Treatment of one psoriatic patient using anti-TNF monoclonal antibody (adalimumab) showed substantial drop (75%) in frequency of NKp44^+^ ILC3 and equivalent increase in population of NCR^−^ ILC3s. This reduction inversely correlated with disease severity (PASI score 21.2 to 13.6) which shows the potential importance of NCR^+^ ILC3 in the pathogenesis of psoriasis.

## Conclusions

In the skin, innate lymphoid cells comprise ILC1, ILC2 and ILC3 populations [41, 46, 47]. Group 2 innate lymphoid cells express CD45, CRTH2 and IL-7Rα while negative for common lineage markers. Bearing receptors for epithelial cytokines and lipid mediators, they produce IL-13, IL-5, IL-4 and IL-9 in response to IL-33, IL-25, TSLP and PGD2 [39, 40]. In the skin, ILC2s express skin homing markers and play a role in type 2 mediated inflammation. Indeed, a higher frequency of ILC2 with an activated phenotype was observed in the lesional skin biopsies of patients with atopic dermatitis and established mouse models of atopic dermatitis supported their contribution to the pathogenesis of this disease [39, 42, 45]. Therefore ILC2s and their activating cytokines or lipid mediators may be new targets for the treatment of atopic dermatitis.

Although research in the field of innate lymphoid cells is moving at a fast pace, many important questions regarding the role of ILC in health and disease still remain unanswered. Detailed interactions of ILC2 with other cell types, including epithelial cells, keratinocytes, fibroblasts, and other cells of the innate and adaptive immune systems would provide a better understanding of the extent of their contribution to homeostatic conditions and disease pathogenesis. Detailed evaluation of signals and mechanisms that regulate ILC activation and inhibition during and after the onset of inflammation and epithelial dysfunction, would help us to identify specific targets for therapeutic interventions.

## Abbreviations

DC: Dendritic cell
NK: Natural killer
ILC: Innate lymphoid cells
TSLP: Thymic stromal lymphopoietin
FALC: Fat associated lymphoid clusters
KLRG1: Killer-cell lectin like receptor G1
COPD: Chronic obstructive pulmonary disease
TCR: T cell receptor
MLN: Mesenteric lymph nodes
GALT: Gut associated lymphoid tissues
ROR: Receptor tyrosine kinase-like orphan receptor.
